# Dual fungal endocarditis in a pediatric dialysis patient: First case of *Aspergillus flavus* and *Candida parapsilosis* co-infection

**DOI:** 10.1016/j.mmcr.2025.100735

**Published:** 2025-09-20

**Authors:** Tasneem Shamsi Basha, Amina Bakro, Hari Pankaj Vanam, Akela Ghazawi, Areej AlGhamdi, Fatima Al Dhaheri

**Affiliations:** a Department of Pediatrics, Sheikh Khalifa Medical City, Abu Dhabi, United Arab Emirates; b Department of Pediatric Nephrology, Sheikh Khalifa Medical City, Abu Dhabi, United Arab Emirates; c Department of Pediatrics, College of Medicine and Health Sciences, Al Ain, Abu Dhabi, United Arab Emirates; d Department of Medical Microbiology and Immunology, College of Medicine and Health Sciences, Al Ain, Abu Dhabi, United Arab Emirates

**Keywords:** Fungal endocarditis, Aspergillus flavus, Candida parapsilosis, Pediatric hemodialysis, End-stage renal disease (ESRD) conflict-zone healthcare

## Abstract

We report the first pediatric case of dual fungal infective endocarditis (IE) caused by *Aspergillus flavus* and *Candida parapsilosis* in a dialysis-dependent child from a conflict-affected region. Diagnosis was supported by galactomannan monitoring, histopathology, and molecular sequencing. Management required early surgery and prolonged dual antifungal therapy. Despite the rarity and severity of such coinfections, the patient survived. This case highlights the importance of early recognition, multidisciplinary care, and aggressive treatment in pediatric fungal IE.

## 1 Introduction

Fungal infective endocarditis (IE) is an uncommon but highly lethal infection, accounting for fewer than 2 % of all IE cases and carrying mortality rates exceeding 50 % [1]. While traditionally associated with immunocompromised states, fungal IE is increasingly recognized in immunocompetent patients with structural or iatrogenic risk factors, such as long-term central venous catheters or prosthetic valves [2].

Pediatric patients with end-stage renal disease (ESRD) on hemodialysis (HD) are especially vulnerable due to chronic catheter use, repeated vascular access, and uremia-related immune dysregulation [3]. *Candida parapsilosis* is a known cause of catheter-associated bloodstream infections due to its biofilm-forming ability on synthetic surfaces [4]. *Aspergillus flavus*, though less common than *Aspergillus fumigatus*, has also been implicated in native valve endocarditis, including in immunocompetent hosts, likely via endothelial invasion following mucosal or vascular injury [5].

Diagnosis is often delayed, especially for *Aspergillus*, due to the low sensitivity of blood cultures. In such cases, histopathology of resected tissue or embolic material remains the gold standard [6], with adjunctive galactomannan (GM) assays offering supportive, but context-sensitive diagnostic information [7].

Here, we report what we believe to be the first documented case of dual fungal IE caused by *A. flavus* and *C. parapsilosis* in a dialysis-dependent, immunocompetent pediatric patient. This case illustrates the convergence of fungal virulence, chronic renal disease, and global health disparities, underscoring the importance of early, multidisciplinary intervention in complex fungal IE.

## 2 Case presentation

A 13-year-old boy with end-stage renal disease (ESRD) secondary to bilateral renal dysplasia, maintained on HD, presented with a three-month history of recurrent fever. His clinical vulnerability was exacerbated by the collapse of the healthcare infrastructure in his war-torn region, including the destruction of the dialysis unit he relied on. The inadequate infection control and poor ventilation at the facility further heightened his risk of infectious complications, underscoring the severity of this case and the importance of thorough evaluation and care.

On admission (Day 0), the patient appeared acutely unwell. His vital signs were notable for a heart rate of 112 beats/min, respiratory rate of 30 breaths/min, blood pressure of 127/86 mmHg, oxygen saturation of 98 %, and a maximum recorded temperature of 38.6 °C. He weighed 32.6 kg and measured 148 cm in height, corresponding to a weight below the 3rd percentile and a height at the 10th percentile for age, consistent with chronic malnourishment. The patient had a pre-existing central venous catheter for HD. According to his mother, the patient had received multiple courses of antibiotics in his home country prior to arrival, though no specific documentation was available.

Initial laboratory investigations revealed severe anemia (hemoglobin 38 g/L), leukocytosis (12.5 × 10^9^/L) with a left shift (neutrophils 80 %, lymphocytes 13 %), and a normal platelet count (154 × 10^9^/L). Inflammatory markers were markedly elevated, including C-reactive protein at 254 mg/L and procalcitonin at 62 ng/mL. Renal function tests were consistent with his known ESRD, showing elevated creatinine (811 μmol/L enzymatic: 789 μmol/L) and cystatin C at 8.08 mg/L. Empiric antimicrobial therapy was initiated following blood culture collection, using vancomycin and ceftazidime. Doses were adjusted based on renal function and serum drug levels. Both antibiotics were discontinued once serial blood cultures remained persistently negative.

**Fig. 1:**
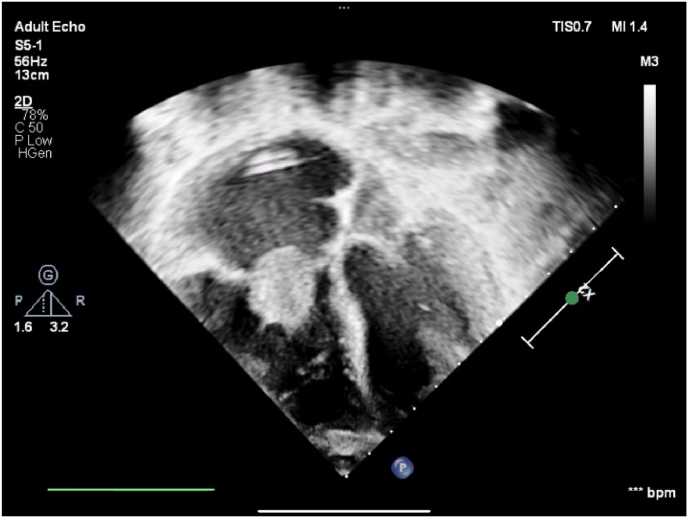
Echocardiogram showing normal cardiac anatomy and function. A 2.5 × 1 cm thrombus is seen in the right ventricle. Tip of a left subclavian catheter is visible in the right atrium.

Echocardiography (ECHO) on admission revealed a right ventricular thrombus measuring 2.5 × 1 cm, not associated with the dialysis catheter tip (Fig. 1). Additional findings included tricuspid regurgitation and a mild pericardial effusion, while biventricular systolic function remained preserved. Imaging confirmed that the subclavian dialysis catheter tip was positioned in the right atrium, with both the carotid and subclavian veins appearing patent. He was promptly transferred to the Pediatric Intensive Care Unit (PICU) and initiated on systemic anticoagulation with heparin. Given the presence of intracardiac thrombus and clinical suspicion for IE, empiric antimicrobial therapy was initiated with Linezolid and Ceftazidime. However, due to persistent fever and rising inflammatory markers, antibiotic coverage was escalated to Meropenem to broaden antimicrobial spectrum and address potential resistant organisms, which was discontinued on Day 16 of admission.

**Fig. 2:**
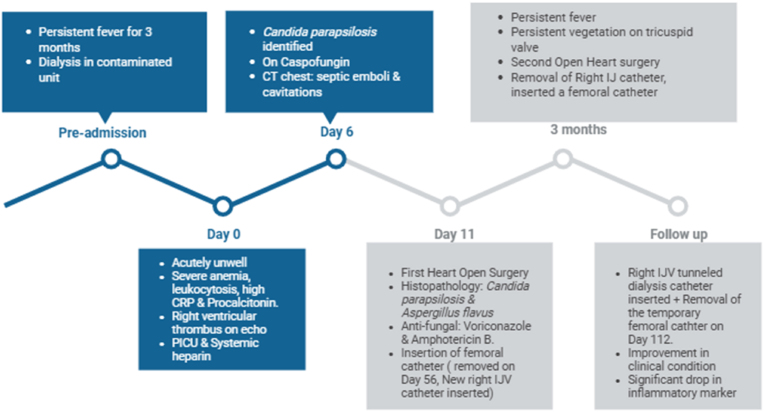
Timeline summarizing key events**,** interventions**,** and outcomes**.**

On Day 4 of admission, blood cultures obtained on Day 2 grew yeast, prompting the initiation of caspofungin (CSF). Treatment was started with a loading dose of 70 mg/m^2^ (70 mg), followed by 50 mg daily for five days, then increased to 70 mg/day in response to the invasive nature of the infection. By day 6, culture identification confirmed *C. parapsilosis*, which demonstrated lower minimum inhibitory concentrations (MICs) to CSF. A summary of key clinical events is illustrated in the timeline below (Fig. 2). A Computed Tomography **(**CT) pulmonary angiogram revealed evidence of both acute and chronic pulmonary emboli, septic pulmonary nodules, and cavitated pulmonary infarcts, consistent with disseminated infection and embolic complications.

Despite appropriate antifungal therapy, serial echocardiograms showed progressive enlargement of the intracardiac thrombus, raising concern for persistent fungal endocarditis. Given the increasing thrombus burden and risk of further embolization, the patient underwent open-heart surgery on day 11 for removal of the tricuspid valve vegetation. A median sternotomy was performed, with complete excision of vegetative material from the right atrium and tricuspid valve, followed by tricuspid valve repair with annuloplasty. Intraoperative findings revealed dense, adherent vegetation on the tricuspid valve and a thickened pericardium, further supporting the diagnosis of invasive fungal endocarditis.

Postoperative ECHO showed no residual intracardiac mass, a stable pericardial effusion, and persistent severe tricuspid regurgitation, necessitating close clinical monitoring. Histopathologic examination of the excised right atrial vegetation was performed. It demonstrated septate hyphae with dichotomous branching, consistent with invasive fungal infection, favoring *Aspergillus*. These findings were confirmed independently by two pathologists. Cultures also grew *Candida* and *Aspergillus* species, supporting a mixed fungal etiology. This prompted the initiation of combined antifungal therapy with oral voriconazole (VRC) and liposomal amphotericin B (AmB). Voriconazole was administered orally at 9 mg/kg/day (285 mg/day). Liposomal amphotericin B was initiated at 3 mg/kg (100 mg) and escalated to 5 mg/kg (160 mg) based on clinical response.

Despite surgical intervention and antifungal treatment, the patient continued to experience persistent fevers and rising inflammatory markers, with procalcitonin levels exceeding 100 ng/mL. His condition was further complicated by progressive septic pulmonary emboli, requiring increasing oxygen support. He also developed pleural and pericardial effusions, necessitating pericardial drainage on postoperative day 12. Notably, cultures from both pericardial fluid and blood remained negative throughout this period.

In the days following drainage, his fever began to resolve, and he showed notable clinical improvement. Given the isolation of *A. flavus*, weekly *Aspergillus* GM monitoring was initiated. The first post-culture reading was elevated at 6.56, with subsequent weekly values fluctuating between 1.98 and 8.04, without a consistent trend.

The patient's immunological workup was unremarkable, with no evidence of significant cellular immune compromise. Lymphocyte subsets were within normal range: CD3^+^ T cells at 2069 cells/μL (73.97 %), CD4^+^ T cells at 1166 cells/μL (43.14 %), and CD8^+^ T cells at 688 cells/μL (25.46 %). Given the concern for chronic granulomatous disease (CGD) as a potential underlying immunodeficiency, a dihydrorhodamine (DHR) oxidative burst test was performed, which returned normal (Stimulation Index = 16.01), effectively ruling out CGD.

Repeated imaging, including CT angiography and whole-body Magnetic Resonance Imaging (MRI), showed reduction in cavitated lesions and embolism-related aneurysmal dilatations. However there were still emergence of new small nodular opacities, possibly still reflecting infection but with less aggressive features. These findings suggest a positive response to therapy, with chronic embolic changes remaining and reduced inflammatory damage. The imaging also revealed a migrated catheter tip lodged in the posterior lower lobe of the lung. The retained catheter fragment was presumed to have originated from a prior central line placed in the patient's home country, where medical care was delivered under resource-constrained conditions in a war-torn setting. While this retained catheter fragment was suspected to be a potential nidus for persistent infection, surgical removal was deferred due to the patient's elevated operative risk.

Throughout the patient's hospitalization, maintaining reliable dialysis access was a persistent challenge due to recurrent infections and multiple surgical interventions. On Day 8 from admission, an infected left-sided hemodialysis catheter was removed. A temporary femoral catheter was inserted on Day 11, in preparation for the first cardiac surgery. This was later replaced with a right internal jugular (IJV) catheter on Day 56. During the second cardiac surgery on Day 97, a new femoral catheter was inserted. Finally, on Day 112, a tunneled right IJV dialysis catheter was placed, and the temporary femoral catheter was concurrently removed. This sequence of catheter placements and exchanges highlights the complexity of vascular access management in patients undergoing repeated surgical procedures and experiencing catheter-related infections.

After three months of systemic antifungal therapy and prior open-heart surgery, the patient continued to experience intermittent fevers and evidence of persistent valvular vegetation. Serial follow-up echocardiography showed normal left ventricular systolic function and acceptable right ventricular systolic function. The mitral valve was competent, but severe tricuspid regurgitation persisted. A residual vegetation measuring 0.5 × 0.5 cm was identified on the anterior tricuspid leaflet, decreased in size from previous evaluations. Additionally, there was trivial pulmonary regurgitation, with unobstructed flow through the main and proximal branch pulmonary arteries. While no pericardial effusion was observed, extracardiac mediastinal fluid had increased in volume, from 2 × 1.1 cm to 4 × 2 cm, compared to prior imaging studies.

Due to the lack of clinical and echocardiographic resolution, the decision was made to proceed with a second open-heart surgery for tricuspid valve replacement. On day 97 a redo sternotomy was performed, which revealed dense adhesions and extensive valvular destruction. The tricuspid valve was severely infected and friable, requiring excision of all three valve leaflets along with involved papillary muscles and surrounding infected tissue. A 25 mm Perimount Magna tissue valve, pre-soaked in VRC, was successfully implanted in the tricuspid position.

**Fig. 3:**
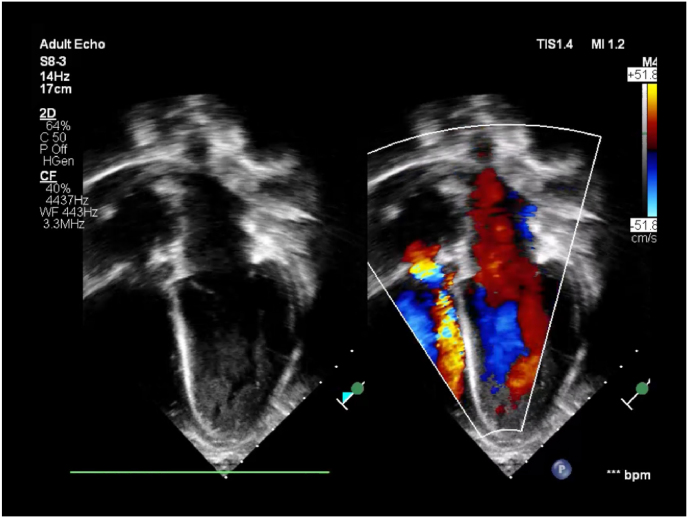
Postoperative follow up echocardiogram showing normal cardiac anatomy. Tricuspid bioprosthetic valve is well positioned with mild stenosis and mild regurgitation. Right ventricular function is mildly to moderately reduced. Trace pericardial effusion is present.

Postoperative ECHO demonstrated moderate tricuspid regurgitation with a gradient of 18 mmHg, normal right ventricular pressures, and a mild paravalvular leak, with no residual vegetations noted. Follow-up echocardiograms showed improvement, with mild to mild-plus regurgitation across the bioprosthetic valve (Fig. 3).

Initial imaging suggested septic pulmonary emboli and cavitated infarcts, which may result from fungal or bacterial infections. The evolution of findings—notably, resolution of cavitated lesions and presence of ground-glass halos—was more consistent with fungal etiology, particularly invasive pulmonary aspergillosis. No bacterial cultures were positive, and response to antifungal therapy supported this interpretation.

While the patient's serum GM levels fluctuated, the highest recorded value reached 9.8. Clinically, he remained hemodynamically stable and afebrile. Persistently elevated galactomannan (GM) levels following valve replacement were thought to reflect retained foci of infection, including a suspected retained catheter tip and chronic embolic lung lesions. A PET scan was requested to evaluate for ongoing disease. Dual antifungal therapy was continued not as prophylaxis, but as active treatment for these residual infectious foci, particularly the inaccessible catheter fragment and ongoing biomarker elevation. These findings supported a continued therapeutic indication. He was discharged home on day 137 XX on oral VRC, with a plan to receive liposomal AmB three times weekly as a short-stay outpatient.

### 2.1 Phenotypic and molecular identification

**Fig. 4:**
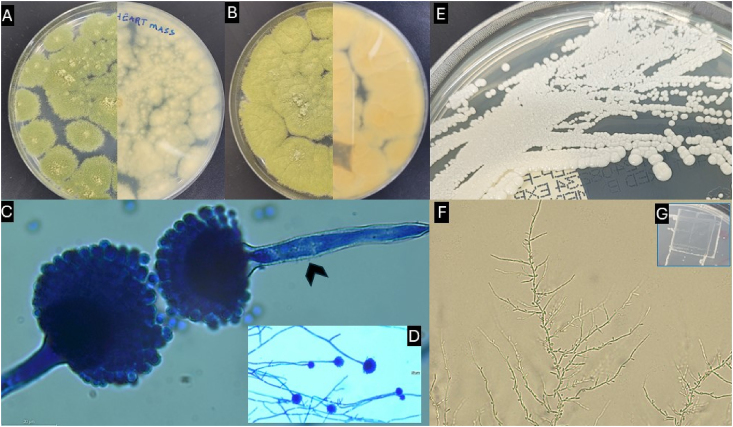
Morphological Features and Colony Attributes**: (A**–**B)** Colony characteristics obverse and reverse of *A. flavus* complex on PDA and SDA-Chloramphenicol. **(C**–**D)** Micromorphological features displaying rough-walled conidiophores (arrowhead) and vesicles with uni- and biseriate phialides radiating across the vesicle surface. (**E**) Colony attributes *C. parapsilosis* on SDA. (**F-G**) Distinct features of *C. parapsilosis* on CMA with Tween 80, including short pencil-like pseudohyphae, oval or round blastospores arranged singly along pseudohyphae. Scale bars: microscopic (10 μm).

Clinical isolates were confirmed at the Fungal Reference Laboratory at UAE University, where quality-checked pure cultures were assigned unique accession numbers and preserved. On Potato Dextrose Agar (PDA), the *Aspergillus* species exhibited white-to-olive-green colonies with a velvety texture on the obverse and a pale-to-yellow reverse. Rough-walled conidiophores terminated in spherical vesicles bearing radiating conidial heads densely covered with phialides. The conidiogenous cells were uni-to biseriate, producing smooth to rough green spherical conidia, confirming placement within the *A. flavus* complex (Section *Flavi*) (Fig. 4A–D). *C. parapsilosis* formed smooth, yeast-like colonies on Sabouraud Dextrose Agar (SDA); on Cornmeal Agar (CMA) with Tween 80, microscopic examination revealed short pencil-like pseudohyphae, oval or round blastospores arranged singly along pseudohyphae, and occasional “giant cells” (Fig. 4E–F). Due to morphological overlap among cryptic species in both implicated genera, the isolates were processed for molecular identification and antifungal susceptibility testing (AFST).

Molecular identification of fungal isolates was conducted using a multi-locus sequencing approach to ensure taxonomic resolution. The internal transcribed spacer (ITS) region of the rDNA was amplified using the universal fungal primers BMB-CRF and ITS4-R [8], allowing for the initial classification of isolates within *Aspergillus* Section *Flavi* and *Candida parapsilosis*. For precise species-level identification, *Aspergillus* isolates were further analyzed by sequencing the β-tubulin (benA) and calmodulin (cmdA) genes, confirming their identity as *A. flavus* sensu stricto [9]. Species verification of *C. parapsilosis* was achieved by sequencing the D1/D2 domain of the large subunit (28S) ribosomal RNA gene [10]. All generated sequences have been deposited in the NCBI GenBank database.

#### 2.1.1 Antifungal susceptibility testing (AFST)

In vitro AFST for *A. flavus* was performed at the Fungus Testing Laboratory (Department of Pathology & Laboratory Medicine, University of Texas Health Science Center, San Antonio, TX, USA) using broth microdilution (BMD) according to CLSI M38-A3 guidelines for filamentous fungi [11]. For *C. parapsilosis*, the Sensititre YeastOne™ Colorimetric Broth Microdilution method (Purelab, Abu Dhabi, UAE) was employed, with results interpreted per EUCAST E. Def 7.4 criteria [12]. The AFST results for *A. flavus* and *C. parapsilosis* are presented in Table 1.

**Table 1:** Minimum inhibitory concentrations (MICs) of antifungal agents.

Antifungal Agent	*Aspergillus flavus* MIC (μg/mL) (CLSI M38-A3)	*Candida parapsilosis* MIC (μg/mL) (Interpreted by EUCAST criteria)
**Amphotericin B (AmB)**	1	≤0.25
**Anidulafungin (AFG)**	≤0.015	Not tested*
**Caspofungin (CSF)**	0.125	0.25
**Micafungin (MCF)**	≤0.015	Not tested*
**Itraconazole (ITC)**	0.5	Not tested
**Posaconazole (PSC)**	0.25	Not tested
**Voriconazole (VRC)**	1	0.12
**Isavuconazole (ISA)**	1	Not tested
**Fluconazole (FLC)**	Not tested	0.5

**Foot note:** AFG and MCF were not tested* for *C. parapsilosis* due to EUCAST recommendations against routine echinocandin testing for this species.

## 3 Discussion

This case highlights the rare occurrence of dual fungal infective endocarditis (IE) caused by *Candida parapsilosis* and *Aspergillus flavus* in a pediatric patient with end-stage renal disease (ESRD), who was immunocompetent but chronically medically complex. While fungal IE is classically associated with immunocompromised states, this case underscores that children with ESRD on hemodialysis remain at substantial risk for invasive fungal infections due to frequent catheter access, prolonged vascular exposure, and uremia-associated immune dysregulation [13]. Fungal endocarditis represents only 1–3 % of all IE cases but carries disproportionately high morbidity and mortality [1].

*Candida* species are the predominant pathogens, particularly *C. albicans* (35–60 %), followed by *C. parapsilosis* (15–41 %) and *C. tropicalis* (10–13 %). *Aspergillus* species, especially *A. fumigatus* and *A. flavus*, account for 20–30 % of fungal IE cases [14,15]. A 2022 systematic review by Meena et al. reported *Candida* (49.6 %) and *Aspergillus* (30 %) as the most frequent fungal pathogens, with prosthetic valve involvement and IV drug use as leading risk factors, and an overall mortality rate of 40 % [16].

Our case builds on prior reports, including Manian et al. [17], and a tertiary-care center retrospective study, both identifying hemodialysis and long-term central venous access as the most common risk factors for fungal IE, with high associated mortality [18].

Polymicrobial fungal IE remains exceedingly rare and clinically challenging. Previous cases of dual fungal or fungal–bacterial endocarditis (e.g., *Burkholderia cepacia*, *Streptococcus sanguinis*) show synergistic virulence and therapeutic resistance, often requiring early dual antifungal therapy and surgical intervention [19].

Echocardiographic findings in fungal IE often reveal large, friable vegetations and peripheral emboli, as seen in over 50 % of cases and in our patient. The diagnosis is further complicated by the frequent negativity of blood cultures in *Aspergillus* infections. In our case, tissue biopsy confirmed the dual infection, fulfilling EORTC/MSG criteria for proven invasive fungal disease [20].

Voriconazole remains first-line therapy for *Aspergillus*, but rising azole resistance such as *A. flavus* strains with *cyp51C* mutations necessitates alternatives like isavuconazole or amphotericin B. In this case, susceptibility to both voriconazole and liposomal amphotericin B was retained (MICs 1 μg/mL). *C. parapsilosis*, though historically less virulent, has emerged as a multidrug-resistant pathogen, especially under regional azole pressure. AFST and sequencing remain essential, as echinocandin resistance often driven by *FKS* mutations can arise despite low MICs [[21], [22], [23], [24]].

Serial galactomannan (GM) monitoring played a crucial role in assessing treatment response. While GM interpretation in non-neutropenic patients remains challenging, its utility in tracking invasive aspergillosis, particularly when blood cultures are negative, is well documented [13,25,26].

Surgical intervention remains a cornerstone of fungal IE management. In this case, the need for a second valve replacement surgery highlights the aggressive nature of the infection and supports data showing improved outcomes with early surgery, particularly in *Aspergillus* IE, where antifungal monotherapy is often inadequate [7].

Mortality in pediatric fungal IE remains alarmingly high, particularly in neonates and children with prosthetic valves or structural heart defects. Table 2 summarizes reported pediatric outcomes, with mortality exceeding 50 %, and reaching 93 % in *Aspergillus* cases. Key risk factors include congenital heart disease, prematurity, and immunosuppression. Early diagnosis, often delayed in mold infections due to blood culture negativity, and aggressive combined therapy are essential [[27], [28], [29], [30], [31], [32], [33], [34]].

**Table 2:** Summary of reported pediatric fungal endocarditis cases in literature [[27], [28], [29], [30], [31], [32], [33], [34]].

Study & Year	Population Studied	Fungal Species (Highlights)	Key Findings	Mortality & Key Outcomes
**Ganesan et al. (2017)** [27]	Systematic review (192 pediatric cases; 1971–2016; 48 % infants, incl. 60 neonates)	**Yeasts (120):** *C. albicans* (72), *C. parapsilosis* (13), *C. tropicalis* (13). **Molds (43):***Aspergillus* spp. (most)	Molds more common in older children; yeasts dominate in infants. Diagnostic delays contribute to high mortality. Cardiac failure = leading cause of death.	**Overall Mortality: 56.25 %**. Neonates (functionally immunocompromised) had high case numbers and mortality.
**Ellis et al. (2001)** [28]	Review (270 fungal endocarditis cases; adult & pediatric)	*C. albicans* (24 %), Non-albicans *Candida* (24 %), *Aspergillus* spp. (24 %), *Histoplasma* spp. (6 %)	Combined surgery + antifungal therapy improves survival. Better survival with *Candida* (vs. molds) and single-valve involvement.	**Overall Mortality: 72 %**. Average patient had 2.5 risk factors (incl. immunosuppression).
**Meshaal et al. (2018)** [29]	Retrospective (374 IE patients; 31 Aspergillus endocarditis (AE); 2005–2016)	*Aspergillus* spp. (100 % of AE subset)	AE associated with prosthetic valves (PVE), healthcare-associated IE (HAE), aortic abscess, and lack of fever.	**AE Mortality:** Higher than non-AE. **Improved AE Survival:** Linked to combination antifungal therapy + surgery.
**Prat et al. (2024)** [30]	Retrospective (32 pediatric IE episodes; 2012–2021; cardiac surgery center)	*C. albicans* (2), *Trichosporon inkin* (2)	Congenital heart disease (CHD) = main risk factor (87.5 %). Prematurity = 25 %. Septic embolism = most common complication (34.4 %).	Fungal IE = 11.1 % of isolates. All 4 fungal cases required surgery.
Burgos **et al. (2009)** [31]	Retrospective (139 pediatric invasive aspergillosis (IA) patients; not exclusively endocarditis)	*A. fumigatus* (52.8 %)	Surgery = only independent predictor of survival for IA. Immune reconstitution best predicts survival (vs. specific antifungals).	**Overall IA Mortality: 52.5 %.** Majority had malignancy/hematopoietic stem cell transplant (HSCT).
**Barst et al. (1981)** [32]	Review (15 pediatric Aspergillus endocarditis cases)	*A. fumigatus*	Most patients had underlying CHD. Blood cultures uniformly negative. Fever/CNS emboli = common presentations.	**Mortality: 93 %** (14/15 died). Survival requires early diagnosis + surgical removal of infected tissue.
**Pasqualotto et al. (2018)** [33]	Case Report (1 immunocompetent child)	*Aspergillus flavus*	Aspergillus endocarditis can occur in immunocompetent children with significant cardiac risk factors (multiple surgeries).	**Favorable outcome** achieved with surgery + antifungals. Emphasizes need for combined therapy.
Levy **et al. (2006)** [34]	Review (30 neonatal candidal endocarditis cases)	*Candida* spp.	Risk factors/outcomes differ from adults. Medical therapy alone may be viable for high-risk neonates unable to tolerate surgery.	**Overall Survival: 73.1 %.** Similar survival: Medical alone (65 %) vs. Medical + surgical (60 %).

Our patient survived beyond six months post-infection, an outcome not previously reported in the pediatric literature for dual *Candida–Aspergillus* IE. This underscores the impact of early multidisciplinary management, surgical intervention, and combination antifungal therapy in improving outcomes for even the most severe fungal endocarditis cases.

Global health inequities also played a critical role in this case. The patient's delayed access to advanced care, limited diagnostic infrastructure, and ongoing exposure to infection risks due to healthcare system collapse exemplify how conflict and fragility exacerbate the burden of complex infections. Enhanced access to diagnostics, antifungal stewardship, and capacity building in conflict-affected settings are urgently needed to address these disparities.

## 4 Conclusion

This case of dual *Aspergillus flavus* and *Candida parapsilosis* endocarditis in a child with ESRD highlights the diagnostic and therapeutic complexity of fungal IE in medically vulnerable but immunocompetent hosts. Successful management required early surgical intervention, antifungal combination therapy, and serial galactomannan monitoring. To our knowledge, this is the first reported pediatric case of dual fungal IE with long-term survival, emphasizing the need for early recognition, multidisciplinary care, and improved access to fungal diagnostics in high-risk settings.

## CRediT authorship contribution statement

**Tasneem Shamsi Basha:** Writing – original draft, Visualization, Data curation. **Amina Bakro:** Writing – original draft, Visualization, Data curation. **Hari Pankaj Vanam:** Writing – original draft, Formal analysis, Data curation. **Akela Ghazawi:** Formal analysis, Data curation. **Areej AlGhamdi:** Writing – review & editing. **Fatima Al Dhaheri:** Writing – review & editing, Validation, Supervision, Project administration, Methodology, Funding acquisition, Conceptualization.

## conflict of interest

N/A.
